# A decade of pharmacovigilance in France: Immune checkpoints join the list of usual suspects for drug‐induced immune haemolytic anaemia

**DOI:** 10.1111/bjh.70152

**Published:** 2025-09-12

**Authors:** Guillaume Brenac, Anne Dautriche, Romane Freppel, Anthony Facile, Bruno Revol, Mathilde Beurrier, Bernard Bonnotte, Sylvain Audia

**Affiliations:** ^1^ Department of Internal Medicine and Clinical Immunology, Constitutive Referral Centre for Adult Autoimmune Cytopenia CHU Dijon‐Bourgogne, Université Bourgogne Europe Dijon France; ^2^ Regional Pharmacovigilance Center of Dijon CHU Dijon‐Bourgogne Dijon France; ^3^ Regional Pharmacovigilance Center of Lyon Hospices Civils de Lyon Lyon France; ^4^ Regional Pharmacovigilance Center of Grenoble CHU Grenoble‐Alpes Grenoble France; ^5^ Regional Pharmacovigilance Center of Nancy CHU Nancy Nancy France

**Keywords:** autoimmune haemolytic anaemia, drug adverse events

To the Editor,

Drug‐induced immune haemolytic anaemia (DIIHA) is a rare adverse drug reaction (ADR) following drug administration with an estimated incidence around 1–2 per million.^1^, ^2^ Different mechanisms of action are involved: (1) drug/antidrug–antibody immune complexes binding non‐specifically to red blood cells (RBC), (2) drug acting as a hapten, modifying RBC antigens, with binding of antibodies only in the presence of the drug and (3) drug triggering auto‐immune haemolytic anaemia (AIHA).^1^ With a direct antiglobulin test (DAT) positive either for IgG with or without complement (C3d) or for C3d only, DIIHA could be misdiagnosed as primary AIHA^3^ and failure to identify the causative drug can lead to inappropriate management of patients. The identification of serum antibodies binding to RBC in the presence of the causative drug is of valuable help, but often restricted to highly specialized laboratories.^1^, ^4^ The drugs most frequently accounting for DIIHA are antineoplastic molecules, antibiotics and non‐steroidal anti‐inflammatory drugs (NSAIDs), but most of the publications lasted from a decade.^2^, ^5^, ^6^

Our aim was to determine whether new molecules accounted for DIIHA during the last years. We thus analysed all cases of DIIHA reported in adults between 2010 and 2021 to the French regional pharmacovigilance centres, a national network assessing the imputability of drugs in ADR reported by clinicians or patients. To avoid exclusion of reports of potential interest, the high‐level terms ‘autoimmune haemolytic anaemia’ and ‘not otherwise specified anaemia’ of the medical dictionary for regulatory activity (MedDRA) were used to identify cases in the national database. To achieve a high level of robustness, we only considered documented cases of haemolytic anaemia (defined as haemoglobin ≤120 g/L with low haptoglobin and increase in other parameters such as lactate dehydrogenase (LDH), free bilirubin or reticulocytes and with a positive DAT and/or drug‐dependent antibodies) with a drug imputability score stated as possible (i.e. intrinsic score ≥2 or ≥3 as defined, respectively, in the 1985 and 2011 updated French causality assessment methods, based on the combination of chronological and semiological scores; Table S1).^7^ Improvement after drug discontinuation followed the French causality assessment method definitions: suggestive (resolution of the adverse reaction, with or without symptomatic treatment, with sufficient time interval and taking into account the pharmacokinetic or pharmacodynamic properties of the drug), inconclusive (irreversible damage or death, unknown outcome, insufficient interval after discontinuation, persistence of ADR without drug withdrawal, persistence of ADR following a single administration) or not suggestive (lack of improvement of reversible ADR despite drug discontinuation with sufficient time interval, complete resolution of ADR despite continuation of medication).^7^ Qualitative data are reported as numbers (percentages), quantitative data as median [1st–3rd quartiles].

Among 975 cases of haemolytic anaemia, 263 occurred in adults and had a high imputability score (Figure 1). A positive DAT or drug‐dependent anti‐RBC antibodies were documented in 81 cases. Thirteen cases were excluded after reviewing the medical history as the diagnosis of DIIHA was reconsidered (*n* = 8) or due to incomplete data (*n* = 5). The median age of the 68 patients finally selected was 64 [50–73] years, with a male‐to‐female ratio of 1.13. Comorbidities were observed in 54.4% (37/68), consistent with solid cancers in 25% (17/68), immune‐mediated diseases in 22% (15/68) and haematological malignancies in 7.4% (5/68) (Figure 1).

**FIGURE 1:**
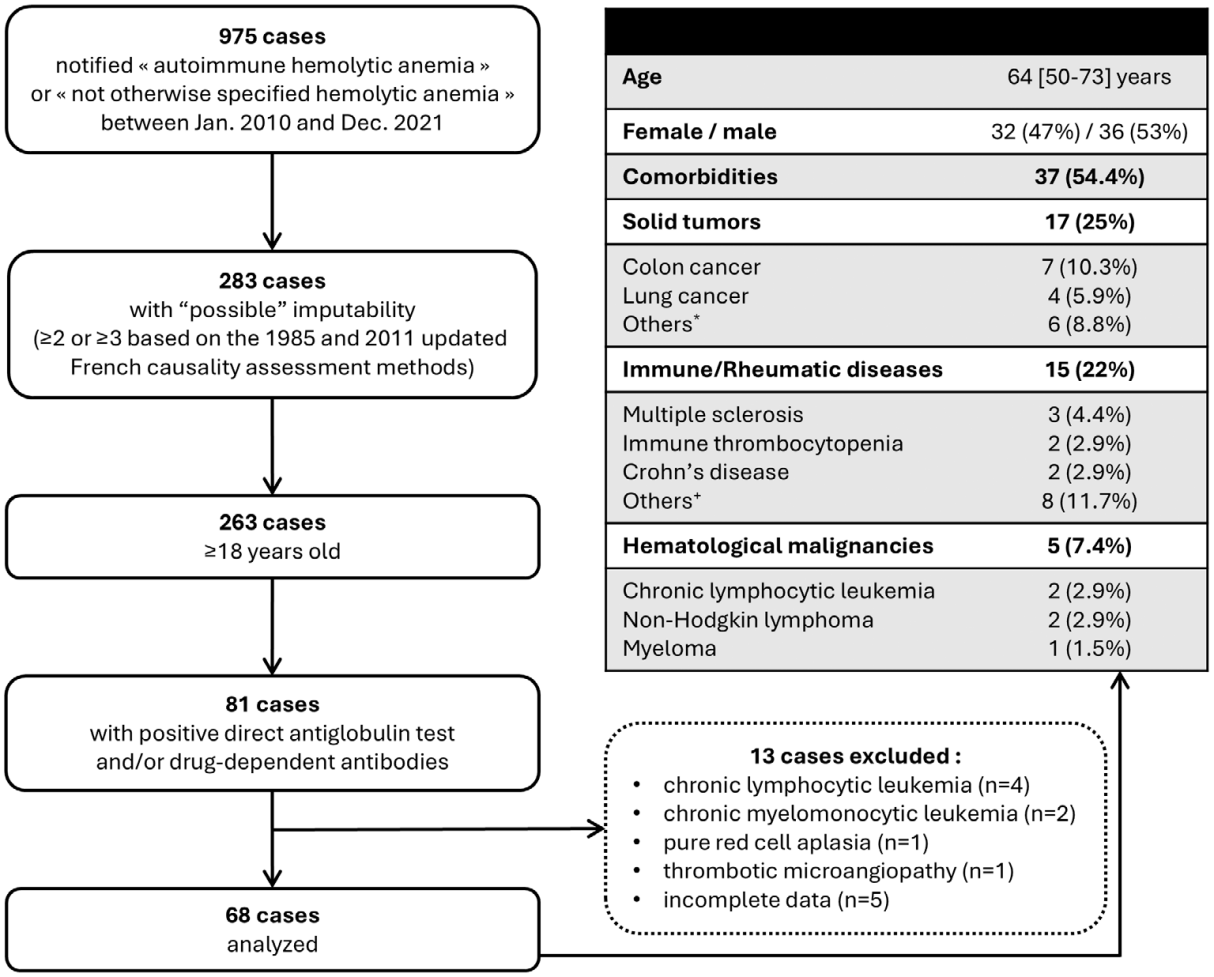
Flowchart and characteristics of patients reported with drug‐induced immune haemolytic anaemia in the French pharmacovigilance database. Data are reported as median [1st–3rd quartiles] or as numbers (frequency). *Other solid tumours were breast, ear–nose–throat, kidney, pancreas, stomach or skin cancers (1 case each), ^+^other immune‐mediated diseases were anti‐neutrophil cytoplasmic antibody (ANCA)‐associated vasculitis, Hashimoto's disease, myasthenia gravis, neutrophilic dermatosis, optical neuromyelitis, psoriatic arthritis, rheumatoid arthritis and Takayasu arteritis (1 case each).

Ten causative drug categories were identified (Figure 2A and Table S2). The most often involved treatments were antibiotics (*n* = 29, 42.7%), headed by piperacillin–tazobactam (41.4% of antibiotics), followed by ceftriaxone (27.6%). Antineoplastic medications were the second most frequent class (*n* = 16, 23.5%), with oxaliplatin accounting for 43.8% of this drug category, while immune checkpoint inhibitors (ICI) represented 25%, with pembrolizumab (18.8%) and nivolumab (6.3%). NSAIDs and intravenous immunoglobulin (IVIG) shared the third position with five cases (7.4%) each. DIIHA was occasionally attributed (≤3 cases/drug classes) to antiparkinsonian agents, immunosuppressants, anticoagulants, anti‐epileptics, antivirals and interferon (Table S2). The median time between drug introduction and DIIHA diagnosis was 1.4 [0.4–4.0] weeks (Figure 2B).

**FIGURE 2:**
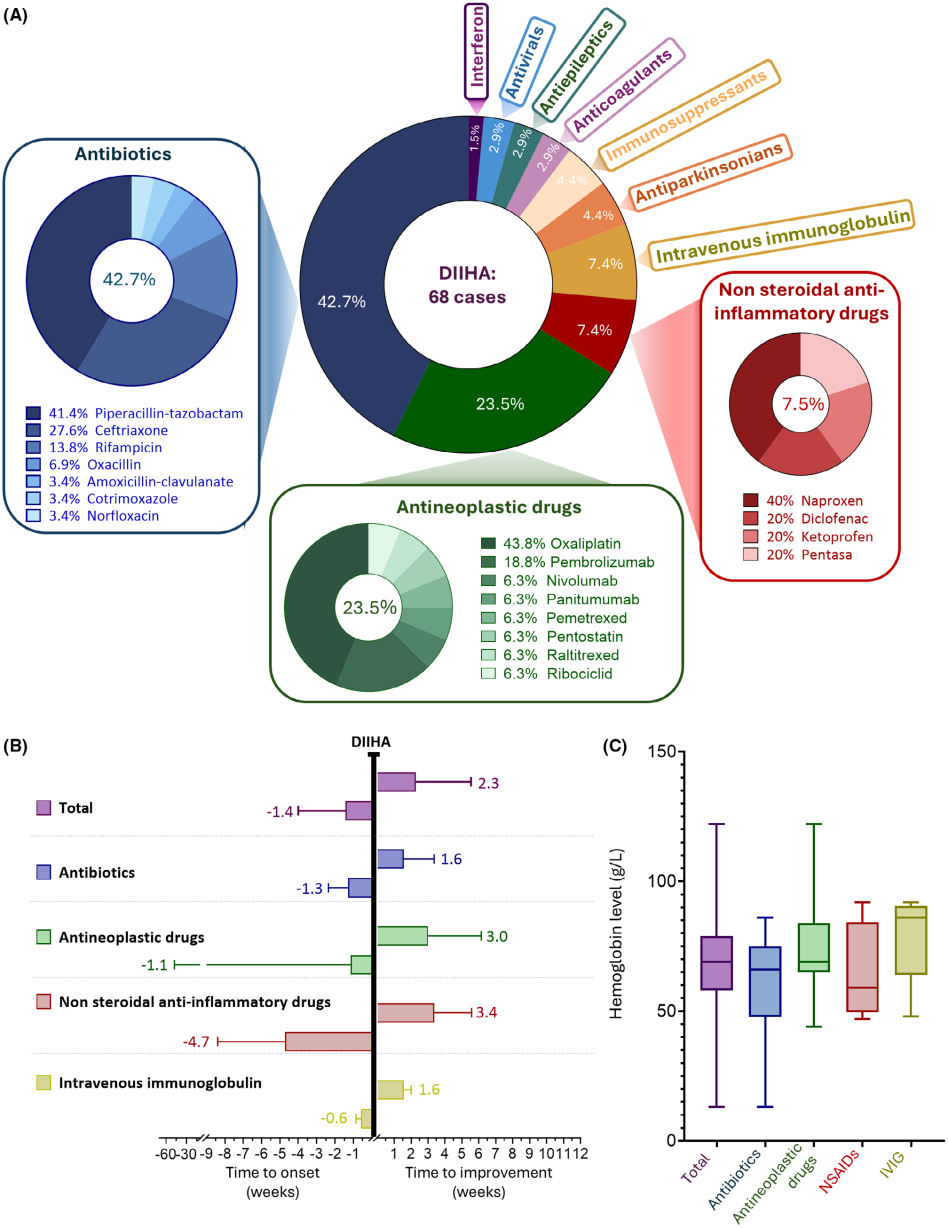
Description of drug‐induced immune haemolytic anaemia cases. (A) Drug classes involved in DIIHA. (B) Median time for DIIHA diagnosis after drug initiation, and median time for improvement after drug discontinuation, for the different drug classes. Histograms represent the median with ranges (bars). (C) Lowest haemoglobin levels for the different causative drug classes. Box and whisker plots showing medians with interquartile (boxes) and minimal and maximal values (bars).

The median lowest haemoglobin level was 69 [58–79] g/L. Although anaemia tended to be less severe with IVIG, there was not a significant difference between drugs (Figure 2C). DAT results were available in 64/68 (94.1%) cases and positive in 62 (96.9%) cases. For other cases, only drug‐dependent anti‐RBC antibodies were reported. DAT specificity was detailed in 61.2% of cases with IgG^+^/C3d^+^ in 17 (27.4%), IgG alone in 16 (25.8%) and C3d alone in 5 (8.0%) cases. Drug‐dependent anti‐RBC antibodies were assessed in 21/68 cases (31%) and detected in 18/21 cases (85.7%), including 12 patients with concomitant positive DAT. Serum drug‐dependent anti‐RBC antibodies were due to piperacillin–tazobactam (*n* = 8), oxaliplatin (*n* = 4), rifampicin (*n* = 2) and in one case each for cotrimoxazole, oxacillin, ritonavir and ceftriaxone.

The causative drug was discontinued in all but one case (67/68, 98.5%). The ADR was considered serious and resulted in hospitalization in 98.5% of cases. Improvement after drug discontinuation, as appraised by the pharmacovigilance physician and/or the clinician managing the patient, was indicated in the chart in 76.9% (50/65) cases and occurred after a median of 2.3 weeks [0.1–17.3] (Figure 2B). RBC transfusion was required in 42.6% (29/68), with a median number of transfusions of 2 [2–3] blood bags. Transfused patients had a lower nadir haemoglobin level (58 g/L [42–66] vs. 77 [69–86]) and were younger (59 years [4–69] vs. 69 [55–73]). Steroids were used in 23/68 (33.8%) cases, without differences between patients treated or not, regarding age, lowest haemoglobin level or transfusion requirement. Death occurred in three cases (4.4%) and was directly related to DIIHA, due to the maintenance of the drug (ceftriaxone) in one case, attributed to both anaemia and sepsis in another and to worsening of kidney and heart failure in a sepsis setting in the last.

A rechallenge with the causative drug was performed in 4/68 (5.9%) cases (secukinumab, piperacillin–tazobactam, nivolumab and natalizumab), leading to the recurrence of DIIHA in all. Rechallenge was not done in 43/68 (63.2%) of cases, while the information was missing for the others. In all cases, the presumed causative drug was contraindicated.

We here show that DIIHA, a situation that might be misdiagnosed as primary AIHA in clinical practice,^1^, ^3^ is a rare but severe ADR with potential life‐threatening outcomes. Most of the patients diagnosed with DIIHA were older than 60 years, 57% of them having comorbidities (solid or haematological neoplasms, autoimmune diseases) as previously reported.^6^ DIIHA occurred shortly after initiation of the drug, with a median time of 10 days.

In accordance with prior data,^1^, ^6^ antibiotics were the first class involved in DIIHA, piperacillin–tazobactam and ceftriaxone accounting for most cases. Antineoplastic drugs represented the second drug category, oxaliplatin being the major drug involved. Interestingly, we observed a growing number of cases due to ICI, accounting for 25% of antineoplastic DIIHA, reflecting their more frequent use and their mechanism of action favouring immune ADR. This is in line with results obtained from the international pharmacovigilance database^8^ and studies dedicated to haematological complications of ICI.^9^, ^10^, ^11^ Of note, we might not have detected all cases secondary to ICI, as DAT could be negative in up to 10%.^9^ In contrast, some classes described as new signals by a recent disproportionality analysis,^8^ such as antidiabetics or cardiovascular system medications, were not reported in our study, which might be related to the difference in study designs. While population‐based studies identify potential associations between drug intake and the occurrence of ADR, our analysis of reports to pharmacovigilance centres provides a stronger causal link, as we only considered cases with a high degree of imputability, that is, with positive DAT or drug‐dependent anti‐RBC antibodies and after careful reviewing of the pharmacovigilance record, thus limiting inclusion of cases due to coincidental association between medication and AIHA. On the other hand, since pharmacovigilance reports are based on self‐reporting, factors such as the date the drug was marketed, the severity of the ADR and the degree of awareness of the ADR may have influenced reporting. Indeed, it has been shown that the reporting peak occurred during the first year of marketing, with a progressive decrease over years and that a majority of ADR were related to unlabelled effects.^12^ Furthermore, after 1 year of marketing, there is a progressive increase in reports of serious adverse effects compared to non‐serious adverse effects.^13^

Although the detection of drug‐dependent anti‐RBC antibodies offers a high level of imputability, they were only screened in a minority of patients (31%). This was most probably due to technical challenges,^4^ as their measurement is limited not only to specialized laboratories but also to possible unawareness of clinicians. Their assessment must be encouraged, as it is an effective method to increase drug imputability.^4^

Formal evidence of causality can also be provided by relapse after rechallenge with the drug, which was rarely attempted in our study, most likely due to the potential severity of the ADR. However, the recurrence that was observed after re‐administration of secukinumab, piperacillin–tazobactam and natalizumab strengthens their imputability in DIIHA.

Apart from the contraindication of the causative drug and supportive care,^14^ it is difficult to draw specific guidelines for the management of DIIHA from our work, particularly concerning the use of immunomodulation with steroids. Indeed, patients were treated by their referring clinician, and the reasons for such management were not detailed in the pharmacovigilance report. The benefit of steroids is debated, but they might be useful in severe cases.^14^ Regarding cases secondary to ICI, there are no formal recommendations as rechallenges have been rarely reported.^15^ In our cohort, the unique rechallenge with nivolumab led to recurrence. However, in the most recent series of 21 DIIHA related to ICI, two patients were re‐administered with the same ICI without relapse of anaemia.^9^ Treatment of cancer being the priority in such cases, rechallenge with ICI should always be discussed, the combination of prophylactic strategies such as rituximab having been proposed.^15^

During the last decade, the most frequently reported drug classes to French pharmacovigilance centres as responsible for DIIHA were antibiotics and antineoplastic drugs, with more cases due to ICI. Drug‐dependent anti‐RBC antibody testing should be encouraged to confirm drug imputability and to avoid misdiagnosis of DIIHA with primary AIHA.

## AUTHOR CONTRIBUTIONS

G.B.: methodology, data curation, formal analysis, writing, reviewing and editing. A.D.: methodology, data curation, formal analysis, validation, reviewing and editing. R.F.: methodology, data curation, reviewing and editing. A.F.: methodology, data curation, validation, reviewing and editing. B.R.: methodology, data curation, validation, reviewing and editing. M.B.: methodology, data curation, validation, reviewing and editing. B.B.: reviewing and editing. S.A.: conceptualization, methodology, supervision, formal analysis, validation, writing, reviewing and editing.

## CONFLICT OF INTEREST STATEMENT

The authors have no conflict of interest to report related to the study.

## Supporting information


Table S1.
Table S2.

## Data Availability

The data that support the findings of this study are available from the corresponding author upon reasonable request.

## References

[1] Garratty G . Immune hemolytic anemia associated with drug therapy. Blood Rev. 2010;24(4–5):143–150. 20650555 10.1016/j.blre.2010.06.004

[2] Mayer B , Bartolmas T , Yurek S , Salama A . Variability of findings in drug‐induced immune haemolytic anaemia: experience over 20 years in a single Centre. Transfus Med Hemother. 2015;42(5):333–339. 26696803 10.1159/000440673 PMC4678312

[3] Zantek ND , Koepsell SA , Tharp DR Jr , Cohn CS . The direct antiglobulin test: a critical step in the evaluation of hemolysis. Am J Hematol. 2012;87(7):707–709. 22566278 10.1002/ajh.23218

[4] Leger RM , Arndt PA , Garratty G . How we investigate drug‐induced immune hemolytic anemia. Immunohematology. 2014;30(2):85–94. 25247618

[5] Garratty G , Arndt PA . Drugs that have been shown to cause drug‐induced immune hemolytic anemia or positive direct antiglobulin tests: some interesting findings since 2007. Immunohematology. 2014;30(2):66–79. 25247621

[6] Garbe E , Andersohn F , Bronder E , Klimpel A , Thomae M , Schrezenmeier H , et al. Drug induced immune haemolytic anaemia in the Berlin case‐control surveillance study. Br J Haematol. 2011;154(5):644–653. 21749359 10.1111/j.1365-2141.2011.08784.x

[7] Miremont‐Salame G , Theophile H , Haramburu F , Begaud B . Causality assessment in pharmacovigilance: the French method and its successive updates. Therapie. 2016;71(2):179–186. 27080836 10.1016/j.therap.2016.02.010

[8] Maquet J , Lafaurie M , Michel M , Lapeyre‐Mestre M , Moulis G . Drug‐induced immune hemolytic anemia: detection of new signals and risk assessment in a nationwide cohort study. Blood Adv. 2024;8(3):817–826. 37782770 10.1182/bloodadvances.2023009801 PMC10874903

[9] Placais M , Laparra A , Maria ATJ , Kramkimel N , Perret A , Manson G , et al. Drug‐induced autoimmune hemolytic anemias related to immune checkpoint inhibitors, therapeutic management, and outcome. Am J Hematol. 2024;99(7):1427–1430. 38642007 10.1002/ajh.27339

[10] Delanoy N , Michot JM , Comont T , Kramkimel N , Lazarovici J , Dupont R , et al. Haematological immune‐related adverse events induced by anti‐PD‐1 or anti‐PD‐L1 immunotherapy: a descriptive observational study. Lancet Haematol. 2019;6(1):e48–e57. 30528137 10.1016/S2352-3026(18)30175-3

[11] Tanios GE , Doley PB , Munker R . Autoimmune hemolytic anemia associated with the use of immune checkpoint inhibitors for cancer: 68 cases from the Food and Drug Administration database and review. Eur J Haematol. 2019;102(2):157–162. 30347480 10.1111/ejh.13187

[12] Haramburu F , Begaud B , Moride Y . Temporal trends in spontaneous reporting of unlabelled adverse drug reactions. Br J Clin Pharmacol. 1997;44(3):299–301. 9296328 10.1046/j.1365-2125.1997.t01-1-00573.x PMC2042840

[13] Moulis G , Sommet A , Durrieu G , Bagheri H , Lapeyre‐Mestre M , Montastruc JL . Trends of reporting of ‘serious’ vs. ‘non‐serious’ adverse drug reactions over time: a study in the French PharmacoVigilance database. Br J Clin Pharmacol. 2012;74(1):201–204. 22257367 10.1111/j.1365-2125.2012.04185.x PMC3394146

[14] Hill QA , Stamps R , Massey E , Grainger JD , Provan D , Hill A . Guidelines on the management of drug‐induced immune and secondary autoimmune, haemolytic anaemia. Br J Haematol. 2017;177(2):208–220. 28369704 10.1111/bjh.14654

[15] Kroll MH , Rojas‐Hernandez C , Yee C . Hematologic complications of immune checkpoint inhibitors. Blood. 2022;139(25):3594–3604. 34610113 10.1182/blood.2020009016 PMC9227102

