# Supplementary material for: A decade of pharmacovigilance in France: Immune checkpoints join the list of usual suspects for drug‐induced immune haemolytic anaemia
Source: Br J Haematol. 2025 Sep 12;207(6):2648–52. doi: 10.1111/bjh.70152 (PMC12710132; doi:10.1111/bjh.70152)
Supplement: Supplementary file 1 — Table S1. Table S2. [file BJH-207-2648-s001.docx]

**Supplemental material**

**Table S1. Intrinsic score (I) as defined in the 1985 and 2011 updated French causality assessment methods, based on the combination of chronological (C) and semiological (S) scores** (Miremont-Salamé *et al.* Therapie, 2016). In our study, only cases with an intrinsic (I) score defined as possible, i.e. ≥2 in the 1985 assessment method or ≥3 in the 2011 assessment method were reviewed (purple boxes).

| **Intrinsic score (I)** | | **1985 French causality assessment** | | | | | | | **2011 French causality assessment** | | | | | | | | | |
| --- | --- | --- | --- | --- | --- | --- | --- | --- | --- | --- | --- | --- | --- | --- | --- | --- | --- | --- |
| **I0** | | C0 | | | | | | | C0 or S0 | | | | | | | | | |
| **I1** | | C1S1, C1S2, C2S1 | | | | | | | C1S1 | | | | | | | | | |
| **I2** | | C2S2, C1S3 | | | | | | | C1S2, C2S1 | | | | | | | | | |
| **I3** | | C2S3, C3S1, C3S2 | | | | | | | C2S2 | | | | | | | | | |
| **I4** | | C3S3 | | | | | | | C1S3, C3S1 | | | | | | | | | |
| **I5** | | - | | | | | | | C2S3, C3S2 | | | | | | | | | |
| **I6** | | - | | | | | | | C3S3 | | | | | | | | | |
| **Chronological score (C)** | | | | | | | | | | | | | | | | | | |
| **Time to onset of the adverse reaction (ADR)** | |  | | | Suggestive | | | | | | Compatible  (neither suggestive nor incompatible) | | | | | | Incompatible | |
| **Rechallenge** | |  | | | R(+) | | R(0) | | R(-) | | R(+) | | R(0) | | R(-) | |  | |
| **Outcome of ADR after drug discontinuation^1^** | | Suggestive | | | C3 | | C3 | | C1 | | C3 | | C2 | | C1 | | C0 | |
|  |  | Inconclusive | | | C3 | | C2 | | C1 | | C3 | | C1 | | C1 | | C0 | |
|  |  | Not suggestive | | | C1 | | C1 | | C1 | | C1 | | C1 | | C1 | | C0 | |
| **Semiological score (S)** | | | | | | | | | | | | | | | | | | |
| **Semiology^2^** |  | Evocative role of the drug  and well-established predisposing factor | | | | | Evocative role of the drug  or well-established predisposing factor | | | | | | No evocative semiology  nor well-established predisposing factor | | | | | |
| **Specific laboratory test^3^** |  | L(+) | | L(0) | L(-) | | L(+) | | L(0) | | | L(-) | L(+) | | | L(0) | L(-) | |
| **Differential diagnosis** | **No other diagnosis** | S3 | | S3 | S1^¤^ | S2^*^ | S3 | | S3 | | | S1 | S3 | | | S2 | S1 | |
|  | **Not investigated** | S3 | | S2 | S1 | | S3 | | S2 | | | S1 | S3 | | | S1 | S1 | |
|  | **Presence of another diagnosis** | S3^¤^ | S2^*^ | S2 | S1 | | S3^¤^ | S2^*^ | S2^¤^ | S1^*^ | | S1 | S3^¤^ | S1^*^ | | S1 | S1^¤^ | S0^*^ |
| R(+): positive rechallenge; R(0): rechallenge not performed; R(-): negative rechallenge; L(+): positive specific laboratory test; L(0): no specific laboratory test performed; L(-): negative specific laboratory test; ^¤^1985 French causality assessment method; ^*^2011 French causality assessment method update; ^1^suggestive : resolution of the ADR following drug discontinuation, with or without symptomatic treatment (with sufficient time interval and taking into account the pharmacokinetic or pharmacodynamic properties of the drug) or following dose reduction for a dose-dependent ADR; inconclusive :irreversible damage or death, unknown outcome, insufficient interval following drug discontinuation, persistence of ADR without drug withdrawal, persistence of ADR following a single administration; not suggestive: lack of improvement of reversible ADR despite drug discontinuation with sufficient time interval, complete resolution of ADR despite continuation of medication; ^2^ evocative because of the pharmacological properties of the drug, the signs suggesting of withdrawal symptoms, the site of the observed ADR; ^3^ specific and reliable lab test or supplementary investigation of the reaction-drug pair, or response to specific antidote. | | | | | | | | | | | | | | | | | | |

**Table S2. Drug classes associated with DIIHA.**

| **Drug classes** | **International nonproprietary name** | **n (%)** |
| --- | --- | --- |
| **Antibiotics, n=29 (42.7%)** | piperacillin-tazobactam | 12 (41.4%) |
|  | ceftriaxone | 8 (27.6%) |
|  | rifampicin | 4 (13.8%) |
|  | oxacillin | 2 (6.9%) |
|  | amoxicillin-clavulanate | 1 (3.4%) |
|  | cotrimoxazole | 1 (3.4%) |
|  | norfloxacin | 1 (3.4%) |
| **Antineoplastic drugs, n=16 (23.5%)** | oxaliplatin | 7 (43.8%) |
|  | pembrolizumab | 3 (18.8%) |
|  | raltitrexed | 1 (6.3%) |
|  | pentostatin | 1 (6.3%) |
|  | pemetrexed | 1 (6.3%) |
|  | ribociclid | 1 (6.3%) |
|  | nivolumab | 1 (6.3%) |
|  | panitumumab | 1 (6.3%) |
| **Non-steroidal anti-inflammatory drugs,**  **n=5 (7.4%)** | naproxen | 2 (40.0%) |
|  | ketoprofen | 1 (20.0%) |
|  | diclofenac | 1 (20.0%) |
|  | pentasa | 1 (20.0%) |
| **Intravenous immunoglobulin, n=5 (7.4%)** | IVIg | 5 (7.2%) |
| **Antiparkinsonians, n=3 (4.4%)** | apomorphine | 2 (66.7%) |
|  | levodopa | 1 (33.3%) |
| **Immunosuppressants, n=3 (4.4%)** | natalizumab | 1 (33.3%) |
|  | secukinumab | 1 (33.3%) |
|  | tacrolimus | 1 (33.3%) |
| **Anticoagulants, n=2 (2.9%)** | dabigatran | 1 (50.0%) |
|  | rivaroxaban | 1 (50.0%) |
| **Antiepileptics, n=2 (2.9%)** | carbamazepine | 1 (50.0%) |
|  | levetiracetam | 1 (50.0%) |
| **Antivirals, n=2 (2.9%)** | ritonavir | 1 (50.0%) |
|  | raltegravir | 1 (50.0%) |
| **Interferon, n=1 (1.5%)** | β1-interferon | 1 (100%) |
